# Identification of a candidate prognostic gene signature by transcriptome analysis of matched pre- and post-treatment prostatic biopsies from patients with advanced prostate cancer

**DOI:** 10.1186/1471-2407-14-977

**Published:** 2014-12-18

**Authors:** Prabhakar Rajan, Jacqueline Stockley, Ian M Sudbery, Janis T Fleming, Ann Hedley, Gabriela Kalna, David Sims, Chris P Ponting, Andreas Heger, Craig N Robson, Rhona M McMenemin, Ian D Pedley, Hing Y Leung

**Affiliations:** Institute of Cancer Sciences, College of Medical, Veterinary and Life Sciences, University of Glasgow, Glasgow, UK; MRC Functional Genomics Unit, Oxford, UK; CR-UK Beatson Institute, Bearsden, UK; Newcastle University, Newcastle, UK; Newcastle-upon-Tyne Hospitals NHS Foundation Trust, Newcastle-upon-Tyne, UK; Cancer Research UK Beatson Institute, Garscube Estate, Switchback Road, Bearsden, G61 1BD UK

**Keywords:** Prostate cancer, Androgen deprivation therapy, Biomarkers, Docetaxel, Cell cycle

## Abstract

**Background:**

Although chemotherapy for prostate cancer (PCa) can improve patient survival, some tumours are chemo-resistant. Tumour molecular profiles may help identify the mechanisms of drug action and identify potential prognostic biomarkers. We performed *in vivo* transcriptome profiling of pre- and post-treatment prostatic biopsies from patients with advanced hormone-naive prostate cancer treated with docetaxel chemotherapy and androgen deprivation therapy (ADT) with an aim to identify the mechanisms of drug action and identify prognostic biomarkers.

**Methods:**

RNA sequencing (RNA-Seq) was performed on biopsies from four patients before and ~22 weeks after docetaxel and ADT initiation. Gene fusion products and differentially-regulated genes between treatment pairs were identified using TopHat and pathway enrichment analyses undertaken. Publically available datasets were interrogated to perform survival analyses on the gene signatures identified using cBioportal.

**Results:**

A number of genomic rearrangements were identified including the *TMPRSS2/ERG* fusion and 3 novel gene fusions involving the ETS family of transcription factors in patients, both pre and post chemotherapy. In total, gene expression analyses showed differential expression of at least 2 fold in 575 genes in post-chemotherapy biopsies. Of these, pathway analyses identified a panel of 7 genes (*ADAM7*, *FAM72B*, *BUB1B*, *CCNB*1, *CCNB2*, *TTK*, *CDK1*), including a cell cycle-related geneset, that were differentially-regulated following treatment with docetaxel and ADT. Using cBioportal to interrogate the MSKCC-Prostate Oncogenome Project dataset we observed a statistically-significant reduction in disease-free survival of patients with tumours exhibiting alterations in gene expression of the above panel of 7 genes (*p* = 0.015).

**Conclusions:**

Here we report on the first “real-time” *in vivo* RNA-Seq-based transcriptome analysis of clinical PCa from pre- and post-treatment TRUSS-guided biopsies of patients treated with docetaxel chemotherapy plus ADT. We identify a chemotherapy-driven PCa transcriptome profile which includes the down-regulation of important positive regulators of cell cycle progression. A 7 gene signature biomarker panel has also been identified in high-risk prostate cancer patients to be of prognostic value. Future prospective study is warranted to evaluate the clinical value of this panel.

**Electronic supplementary material:**

The online version of this article (doi:10.1186/1471-2407-14-977) contains supplementary material, which is available to authorized users.

## Background

The mainstay of treatment for “incurable” locally-advanced/metastatic prostate cancer (PCa) is androgen deprivation therapy (ADT) [1], however after ~2-3 years the disease becomes castration-resistant (CRPCa). Historically, patients with CRPCa exhibited a median survival of less than ~18 months, although this has improved since the advent of novel chemo- and endocrine therapies [2]. The anti-mitotic agent docetaxel was the first chemotherapeutic agent to demonstrate a significant survival advantage for patients with CRPCa [3, 4]. Docetaxel stabilizes microtubules, thereby interrupting microtubule dynamics (including the mitotic spindle) causing mitotic arrest and accumulation of cells in G2/M (due to failure chromosome segregation and cytokinesis) and apoptosis [5, 6].

Early trials demonstrated an overall median ~2-3 month survival advantage for docetaxel-based therapies over standard treatments for CRPCa [3, 4], supporting its recommendation as first-line standard of care for CRPCa [1]. However, only ~50% of patients with CRPCa will respond to docetaxel, and the modest survival advantage is at the cost of significant toxicity [3, 4]. Recently, docetaxel plus ADT have been found to confer no statistically-significant survival advantage over ADT alone for non-CRPCa (i.e. hormone-naïve disease), despite an improvement in clinical and biochemical progression-free survival [7].

An understanding of the biology of *de novo* and acquired chemo-resistance to docetaxel (and other agents) in PCa with in-parallel biomarker discovery will help to identify patients who will not benefit from treatment prior to exposure, thereby avoiding unnecessary toxicity and guiding more effective therapeutic options. Aided by technological advances such as next generation sequencing which facilitate whole genome and transcriptome analyses, molecular profiling of pre- and post-treatment tumour samples may help to identify the mechanisms of drug action and link specific gene amplifications and mutations or expression changes to clinical chemo-sensitivity or -resistance patterns [8].

Previously-published transcriptome-wide analyses of docetaxel action and chemo-resistance in PCa have utilised microarrays for assessment of pre- and post-extirpative surgical specimens [9, 10] and *in vitro* cell lines [3, 11–13]. However, these studies are limited by the inherent bias and quantitative nature of microarray data [14]. We performed *in vivo* transcriptome profiling by next generation RNA sequencing (RNA-Seq) of pre- and post-treatment transrectal ultrasound (TRUSS)-guided prostatic biopsies from patients with newly-diagnosed locally-advanced/metastatic non-CRPCa treated with docetaxel chemotherapy plus ADT.

## Methods

### Patient samples

Patient samples for gene expression analysis (RNA-Seq) were collected as part of the GenTax (Tumour profiling in an open-labelled, two-arm study investigating the tolerability and efficacy of Taxotere in patients with hormone-naïve high-risk prostate cancer) study by Newcastle upon Tyne Hospitals National Health Service (NHS) Foundation Trust [15]. All patients with a clinical suspicion of advanced PCa were subjected to TRUSS-guided prostatic biopsy (BK Medical, 8818) for histopathological assessment by Gleason Sum score [16] of Haematoxylin and Eosin (H&E)-stained tissue. Radiological staging investigations were performed according to national guidelines [17]. Patient eligibility criteria were cT3/T4 [18] PCa, Prostate Specific Antigen (PSA) ≥50 ng/ml or Gleason Sum score ≥8, or metastatic disease to be commenced on ADT. Further eligibility for study inclusion were Karnofsky Performance status (KPS) Score [19] ≥ 70%; a life expectancy of ≥ 3 months; and adequate haematological, hepatic, and renal function. All patients received ADT, which consisted of the goserelin 3.6 mg on a q28-day schedule with anti-androgen “flare” protection and 6 cycles of docetaxel (Taxotere^®^) 75 mg/m^2^ on a q21-day schedule [15]. Further material for RNA-Seq was taken by TRUSS-guided biopsy prior to commencement of chemotherapy and again at ~22 weeks following initiation of treatment. Biopsies were specifically taken from tumour-rich areas of the prostate, where typically over 60% of the initial diagnostic cores taken were occupied by tumour. All patient material was anonymized and stored at -80°C. Serum PSA was measured ~3-weekly until ~22 weeks and then 3-monthly, and repeat radiological staging undertaken at ~6 months after diagnosis for patients with N+ and/or M+ disease to assess the radiological response. PSA progression was defined as two consecutive rises in PSA above nadir at least 2 weeks apart, although whether patients subsequently fulfilled the European Association of Urology (EAU) criteria for castration resistant PCa disease [1] is not known. Written informed consent to participate was obtained from all subjects. Ethical approval was granted from the local research and ethics committee (Northumberland, Tyne and Wear NHS Strategic Health Authority Local Research Ethics Committee Ref: 2003/11).

### RNA extraction and RNA-Seq

Patient samples for RNA-Seq were analysed as previously described [20]. Total RNA was extracted from pre- and post-treatment samples using the RNeasy Mini Kit (QIAgen, 74104) according to manufacturer’s instructions. The NanoDrop 2000 spectrophotometer (Thermo Scientific) and 2100 Bioanalyzer (Agilent) were used to assess RNA quantity and quality, with calculation of RNA integrity number (RIN) [21]. Samples were included for RNA-Seq if RIN > 6 and total RNA > 500 ng. Illumina RNA-Seq was performed according to manufacturer’s instructions, with cDNA sample library normalization using the Illumina DSN (Duplex-specific Nuclease) protocol prior to cluster generation and library sequencing on the HiSeq™ 2000 (Illumina) with a paired-end sequencing strategy. The read length was set at 90 nt with an expected library size of 200 bp.

### Bioinformatics

The FastQC package (http://www.bioinformatics.babraham.ac.uk/projects/fastqc) was used to assess the quality of raw reads, which were then mapped to human genome assembly hg19 using TopHat version 1.4.1 [22] with a junctions library derived from Ensembl version 68. Quality control was performed on all samples by examining the following parameters: (a) the percent of reads uniquely mapping to the genome; (b) the percent of reads mapping to known protein coding sequence; (c) the number of exon junctions identified; (d) the percent of spliced reads; and (e) the number of genes with 90% base coverage (Additional file 1: Table S1). TopHat-Fusion version 0.1.0 [23] was used to identify gene fusions. HTSeq version 0.5.3 (http://www.huber.embl.de/users/anders/HTSeq) was used to identify differentially-expressed genes by counting the number of reads mapping to each gene from Ensembl version 68. The TMM method was used to normalise read counts and differential expression tested for using a paired generalized linear model design with the Bioconductor version 2.11 edgeR package [24]. The Circos plot was generated using RCircos version 1.1.2 [25]. Correlations were identified using Pearson’s product moment correlation coefficient (*p* < 0.05). Enriched KEGG (Kyoto Encyclopedia of Genes and Genomes) pathways [26] were identified by downloading gene pathways associations and testing each pathway for enrichment in significantly up- and down-regulated genes (FDR < 0.05) with a transcript length-corrected Wallenius approximation as implemented by the GOSeq package for Bioconductor 3.0 [27]. Pathways were deemed to be enriched if the enrichment over background was at least 2-fold and the FDR < 0.05. Gene lists were uploaded to cBioPortal (http://www.cbioportal.org) [28, 29] to study gene expression changes in all prostate tumours with mRNA expression data (n = 150) from the Memorial Sloan Kettering Cancer Center (MSKCC) Prostate Oncogenome Project dataset [30] using a mRNA Z-score threshold of ± 1.6 as compared with normal prostate samples. Genes altered in a significant number of tumours (>25%) were considered for associations with disease-free survival though the cBioPortal software using the Kaplan–Meier method with log rank testing with *p* < 0.05 taken to indicated statistical significance. Raw sequencing data have been deposited at Gene Expression Omnibus (http://www.ncbi.nlm.nih.gov/geo/) under accession number GSE51005 and all details are MIAME compliant.

## Results

### The transcriptomic landscape of docetaxel chemotherapy plus ADT in PCa

Next generation RNA sequencing (RNA-Seq) was performed on 12 paired pre- and post-docetaxel plus ADT samples from 6 patients with locally-advanced/metastatic PCa (Table 1). The post-treatment samples from Patients 2 and 3 performed markedly worse on multiple quality control measures, and so all samples from both patients were excluded from further quantitative expression analysis (Additional file 1: Table S1). The remaining 8 samples matched our previously-published dataset on the ADT-only control arm of the GenTax study [20] on two key quality control measures: At least 50 million 90 bp paired-end reads were obtained per sample with at least 40% coverage of transcripts sequenced (Additional file 1: Table S1).

**Table 1:** **Patient demographics of samples for RNA-Seq following docetaxel chemotherapy plus ADT**

Patient	KPS	GSS	T	N	M	iPSA (ng/ml)	nPSA (ng/ml) (% iPSA)	PFS (days)
1	90	9	4	1	1	8.76	0.09 (1.0)	317
2^*^	90	7^$^	3b	1	0	8.44	1.3 (15.4)	105
3^*^	90	8	3b	0	0	25.8	0.19 (0.7)	834
4	90	7	3b	1	1	80.8	0.08 (0.1)	588
5	90	9	3b	0	0	2.85	0.08 (2.8)	N/P
6	90	7	3b	1	1	636	0.1 (0.0)	N/P

All patients exhibited a response to docetaxel plus ADT prior to second TRUSS-guided biopsy as determined by a fall in levels of serum PSA. The mean time to second TRUSS-guided biopsy was 156 ± 37 days. ^*^Samples removed from RNA-seq analysis. ^$^Tertiary Gleason grade 5. (KPS = Karnofsky Performance Status; GSS = Gleason Sum Score; iPSA = initial PSA value at diagnosis; nPSA = nadir PSA value prior to second TRUSS-guided biopsy; PFS = biochemical progression-free survival; N/P = not yet progressed).

**Figure 1:**
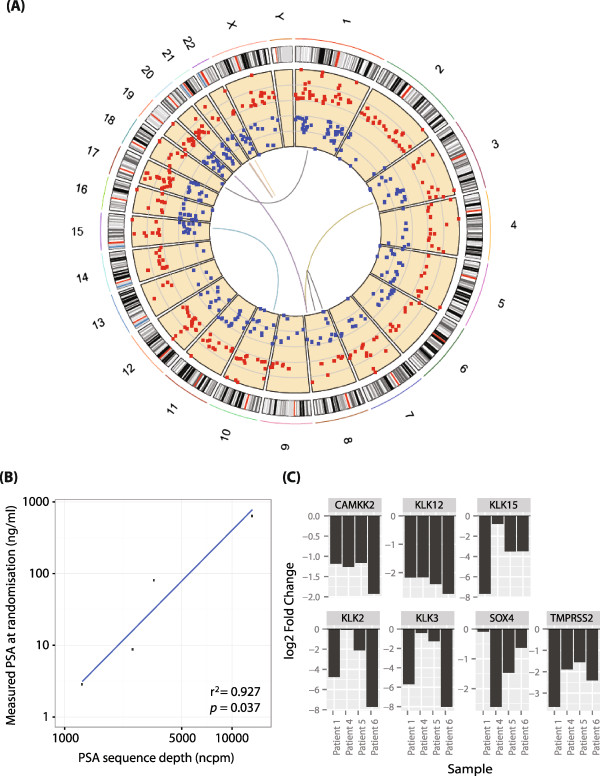
**Differential expression of androgen-regulated genes in response to docetaxel chemotherapy plus ADT. (A)** Circos plot [25] of the transcriptomic landscape of docetaxel chemotherapy plus ADT in PCa. The outer ring shows chromosome ideograms with labelled chromosome identities. The scatter plot shows up- (Red) and down- (Blue) regulated genes. Gene fusions are shown as coloured arcs linking two genomic loci. **(B)** Log-log plot demonstrating correlation between *KLK3* (encodes PSA) mRNA expression levels (X-axis) normalized by trimmed means of M-value (TMM) in normalized counts per million (ncpm) and serum PSA levels (ng/ml) (Y-axis) (r^2^ = 0.927; *p* = 0.037). **(C)** Expression of known androgen-regulated genes (Log_2_ fold change ≥ 2; FDR < 0.05) following docetaxel plus ADT.

Genomic rearrangements involving ETS-family transcription factors are implicated in PCa with the most common gene fusion product *TMPRSS2/ERG* reported in >50% cases [31]. We searched for expression of transcripts derived from such gene fusions in our datasets. We observed the intra-chromosomal *TMPRSS2/ERG* gene fusion product in only the pre-treatment sample from 1 patient (Patient 3), which was actually excluded from the quantitative expression analysis (Additional file 2: Table S2). However, we observed 3 further novel intra-chromosomal gene fusions: two products were derived from a fusion between *DOPEY2* and *ERG* genes within chromosome 21 (Fusion event 7), and 2 different gene fusions were observed within chromosome 22 (Fusion events 3 and 4) (Figure 1A). A further five novel fusion transcripts were identified (Figure 1A and Additional file 2: Table S2). In three patients, identical inter- and intra-chromosomal gene fusions (*CCNY*/*LRCC49*, *PVT1*/*CPNE4*, and *DOPEY2*/*ERG*) were identified in both pre- and post-treatment samples.

Across the genome, we observed a total of 298 genes up-regulated and 277 genes similarly down-regulated at least 2-fold (False Discovery Rate [FDR] <0.05) in response to docetaxel plus ADT (Figure 1A, Table 2 and Additional file 3: Table S3). The levels of expression of *KLK3*, which encodes PSA (Prostate Specific Antigen), detected by RNA-Seq of the docetaxel plus ADT arm correlated as expected with serum PSA levels (r^2^ = 0.927; *p* = 0.037) (Figure 1B). A number of other known androgen-regulated genes (including those encoding kallikreins) were also consistently down-regulated in the docetaxel plus ADT arm (Figure 1C) suggesting that ADT in combination with docetaxel had the expected action on androgen-regulated gene expression.

**Table 2:** **Differentially-expressed genes following docetaxel chemotherapy plus ADT versus ADT alone**

Gene set	Tax		ADT	
	Up	Down	Up	Down
Protein Coding	298	277	774	755
Non-Coding	7	15	35	116

Numbers of protein coding and non-coding genes differentially expressed at least 2-fold after ADT with FDR < 0.05. (ADT = androgen deprivation therapy; Tax = docetaxel + ADT).

**Figure 2:**
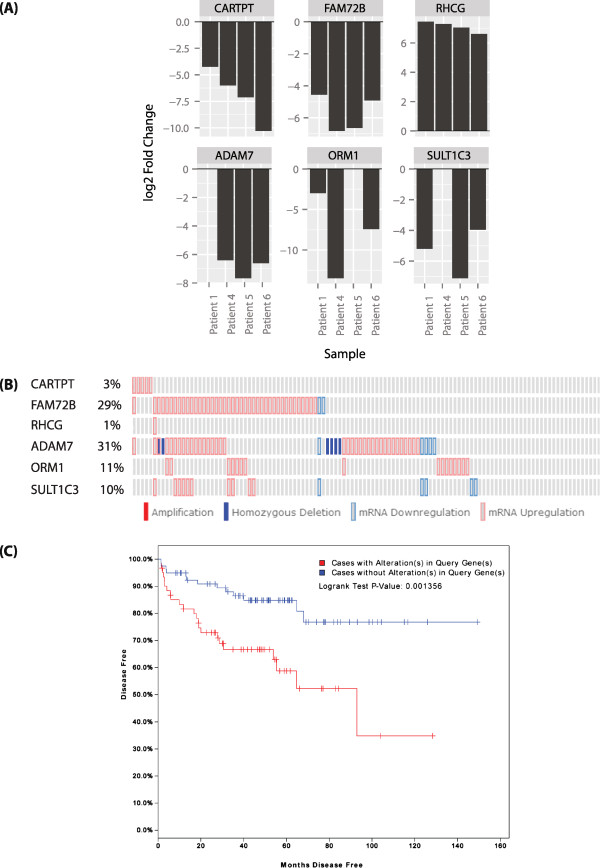
**Differential expression of genes affected by docetaxel chemotherapy plus ADT. (A)** Log_2_ fold change of 6 of the 10 top-ranking differentially-expressed genes (Log_2_ fold change ≥ 2/≤ - 2; FDR < 0.05) consistent in the direction of expression changes in at least 3 out of 4 individual patients. **(B)** Matrix heatmap generated using cBioPortal [28, 29] showing alterations in expression of 6 of the top 10 differentially-regulated genes (exhibiting consistent expression changes in at least 3 out of 4 patients in the present study) in the MSKCC Prostate Oncogenome Project dataset [30]. **(C)** Kaplan Meier plot showing the survival curves of patients in the MSKCC Prostate Oncogenome Project dataset with and without alterations in expression of *FAM72B* and *ADAM7* (*p* = 0.001).

Based on the full gene list (Log2 fold change ≥2/≤ - 2; FDR < 0.05) (Table 2 and Additional file 3: Table S3), we ranked genes according to the magnitude of their fold changes, regardless of whether they were up- or down-regulated. The 10 top-ranking genes differentially-regulated by docetaxel plus ADT were arbitrarily selected (range of fold changes -9.96 to 9.86) for further downstream knowledge-based validation. From these 10 genes, we selected genes that exhibited expression changes consistent in direction in at least 3 out of 4 patients. We identified 6 differentially-expressed genes (Figure 2A) including *ORM1*, which had the highest average level of differential expression of all transcripts in our dataset (Log_2_ fold change = -9.96; FDR < 0.05). This gene encodes an acute phase plasma protein that has been identified as a putative biomarker of chemo-resistance to docetaxel and doxorubicin in breast cancer [13].

Using cBioPortal [28, 29], we interrogated the MSKCC Prostate Oncogenome Project dataset (n = 150) [30] for changes in expression of the above 6 genes in treatment-naive prostate tumours as compared with normal controls. We observed alterations in expression of all 6 genes, with *FAM72B* and *ADAM7* exhibiting significant alterations (Figure 2B). Survival analysis identified a statistically-significant reduction in disease-free survival of patients with tumours exhibiting alterations in expression of this geneset (*p* = 0.023) (Additional file 4: Figure S1A) which was lost when *FAM72B* and *ADAM7* were removed from the geneset (*p* > 0.05) (data not shown). Using only *FAM72B* and *ADAM7*, survival analysis demonstrated a statistically-significant disease-free survival advantage in patients with no alterations in gene expression (*p* = 0.001) (Figure 2C). Taken together, these data suggest that alterations in expression of *FAM72B* and *ADAM7* are associated with early treatment relapse and hence may be biomarkers with prognostic value in treatment-naïve PCa.

### Pathway analyses of gene expression changes in response to docetaxel chemotherapy and ADT

**Figure 3:**
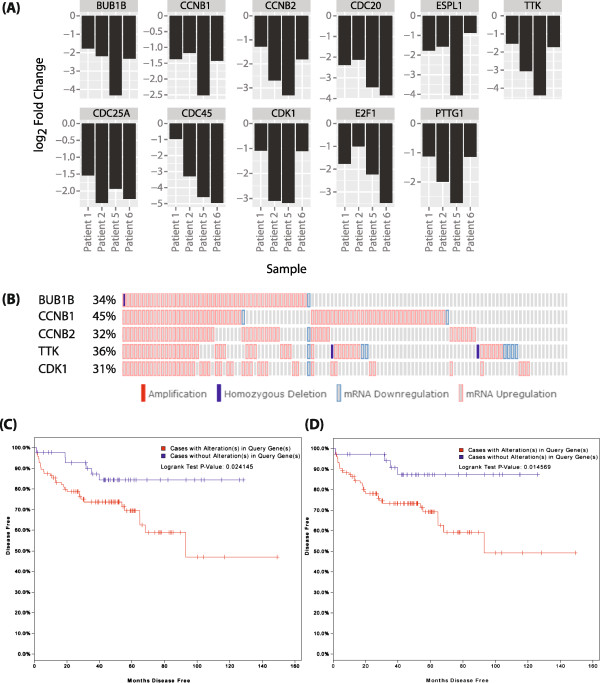
**Pathway analyses of gene expression changes in response to docetaxel chemotherapy with ADT. (A)** Log_2_ fold change of genes enriched (enrichment > 2-fold; FDR < 0.05) within the KEGG (Kyoto Encyclopedia of Genes and Genomes) [26] term “Cell Cycle” following docetaxel plus ADT treatment. **(B)** Matrix heatmap generated using cBioPortal [28, 29] showing alterations in expression of 5 genes from within the KEGG term “Cell Cycle” (*BUB1B*, *CCNB1*, *CCNB2*, *TTK*, and *CDK1*) in the MSKCC Prostate Oncogenome Project dataset [30]. **(C)** Kaplan Meier plot showing the survival curves of patients in the MSKCC Prostate Oncogenome Project dataset with and without alterations in expression of the 5 cell cycle-related genes (*p* = 0.024). **(D)** Kaplan Meier plot showing the survival curves of patients in the MSKCC Prostate Oncogenome Project dataset with and without alterations in expression of the genes in the candidate biomarker panel (*ADAM7*, *FAM72B*, *BUB1B*, *CCNB*1, *CCNB2*, *TTK* and *CDK1*) (*p* = 0.015).

To identify biological pathways perturbed by combined docetaxel chemotherapy with ADT, we performed an enrichment analysis on our lists of up- and down-regulated genes (FDR < 0.05) using 3 different pathways analysis tools: the KEGG (Kyoto Encyclopedia of Genes and Genomes) database [26]; IPA “Core Analysis” function; and Metacore (Figure 3 and Additional file 5: Figure S2, Additional file 6: Table S4 and Additional file 7: Table S5). The KEGG terms “Cell Cycle” (n = 11/124; enrichment = 5.89-fold; FDR = 0.0014) and “Steroid Biosynthesis” (n = 5/19; enrichment = 17.63-fold; FDR = 0.0014) were enriched greater than 2-fold in the down-regulated gene list (Additional file 6: Table S4), while no pathways were significantly enriched in the up-regulated gene list. Genes within the KEGG term “Cell Cycle” included the key positive cell cycle regulators *CCNB1*, *CCNB2*, *CDK1* and *CDC25A* (Figure 3A and Additional file 8: Table S6), the expression of which was down-regulated following docetaxel plus ADT. The Ingenuity Pathway Analysis “Core Analysis” function also identified the “Cell Cycle” as the highest-ranking network containing clusters of docetaxel and ADT-regulated genes (Additional file 5: Figure S2A and Additional file 7: Table S5). Metacore analysis of docetaxel and ADT-regulated genes identified Cell cycle “The metaphase checkpoint” as the 2^nd^ top enriched pathway after Cytoskeleton remodelling “Keratin filaments”, which is consistent with the known actions of docetaxel (Additional file 5: Figure S2B).

The observed enrichment for cell cycle-related genes, including down-regulation of expression of positive regulators of cell cycle progression, is in keeping with the known actions of docetaxel *in vitro* on the induction of G2/M arrest [5]. In the light of evidence suggesting that androgen withdrawal may diminish docetaxel-induced apoptosis *in vitro* [32], we wished to ensure that our *in vivo* observations were consistent with the mechanism of action of docetaxel *in vitro* in the absence of androgens. We used the LNCaP PCa cell line grown in steroid-depleted medium as a model for non-CRPCa treated with ADT. Reassuringly, we observed statistically-significant induction of G2/M arrest (*p* < 0.05) following treatment with docetaxel (at 10 nM, 100 nM or 1 μM doses) (Additional file 5: Figure S2C).

Finally, we used cBioPortal [28, 29] to interrogate the MSKCC Prostate Oncogenome Project dataset [30] for changes in expression of the genes enriched within the KEGG term “Cell Cycle” in clinical PCa and observed alterations in expression of all genes in a large (78%) proportion of cases (Additional file 5: Figure S2D), suggesting that expression of these transcripts is associated with prostate tumourigenesis. Survival analyses did not identify any statistically-significant associations between disease-free survival time in patients with tumours exhibiting alterations in expression of these genes as compared with patients with tumours exhibiting no alterations in expression (*p* > 0.05) (data not shown). However, when genes exhibiting alterations in high (>25%) proportion of tumours only were included in this geneset (Figure 3B), we observed statistically-significant reduction in disease-free survival of patients with tumours exhibiting alterations in expression of this geneset (*p* = 0.024) (Figure 3C). Using a combined geneset of these 5 remaining cell cycle-related genes (*BUB1B*, *CCNB*1, *CCNB2*, *TTK* and *CDK1*) as well as *ADAM7* and *FAM72B*, we also observed a statistically-significant reduction in disease-free survival of patients with tumours exhibiting alterations in gene expression (*p* = 0.015) (Figure 3D). Our observations suggest that these 7 genes in combination could form a panel of biomarkers associated with early relapse from treatment in clinical PCa.

## Discussion

To the best of our knowledge, our study is the first “real time” *in vivo* RNA-Seq-based transcriptome analysis of clinical PCa from pre- and post-treatment TRUSS-guided biopsies of patients treated with docetaxel chemotherapy plus ADT. The limitations of our study include a targeted TRUSS-guided needle-core biopsy strategy that may result in heterogeneous tissue sampling with variable cellularity and small sample numbers due to the high quality RNA required for RNA-Seq (RIN > 6 and total RNA > 500 ng). Despite using fresh-frozen tissue samples, the high sample attrition rate (33%) from analyses prevented more meaningful clinical outcomes, such as treatment response, to be extrapolated from our results. Nonetheless, we clearly demonstrate the feasibility of this *in vivo* approach to obtain informative transcriptomic data from small tissue samples pre- and post-treatment with cytotoxic chemotherapy. As tissue sample processing and RNA-Seq methodologies are further refined, it may become possible to obtain reliable sequencing information from low input and/or degraded clinical samples [33].

The transcriptomic landscape of PCa includes gene fusion products as a result genomic rearrangements [31]. We observed transcripts derived from the commonly-reported *TMPRSS2/ERG* gene fusion as well as other inter- and intra-chromosomal gene fusions. Incorporating different samples from our previously-published RNA-Seq dataset from the same study cohort [20], we observed transcripts arising from the *TMPRSS2/ERG* fusion in 28% of all pre-treatment samples. These observations are comparable to the frequency of *TMPRSS2/ERG* fusions reported in Caucasian populations [34] as well as in an Asian cohort analysed by RNA-Seq [35].

Our analysis of docetaxel plus ADT-driven gene expression changes identified two differentially-regulated genes *ADAM7* and *FAM72B*, which were also mis-regulated in a large proportion of prostate tumours from a large cohort of different patients and associated with shorter disease-free survival after treatment. Additionally, we identified enrichment for cell cycle-related genes, including the down-regulation of expression of some positive regulators of cell cycle progression ~4 weeks after the final cycle of docetaxel chemotherapy. Our observations were somewhat reassuring, as docetaxel in combination with ADT *in vivo* appears to exhibit an expected mechanism of action on cell cycle progression. Furthermore, we demonstrated that androgen withdrawal did not affect the dose-dependent induction of G2/M by docetaxel *in vitro*. Taken together, our data suggest a persistent anti-tumourigenic effect of docetaxel in combination with ADT *in vivo*. However the longevity of this response may be limited, as a previous study of docetaxel-treated tumours identified persistent PCa several months after treatment [36].

Finally, we identify a biomarker panel of 7 genes (*ADAM7*, *FAM72B*, *BUB1B*, *CCNB*1, *CCNB2*, *TTK* and *CDK1*), which included a cell cycle-related geneset, that was not only mis-regulated in a significant proportion of treatment-naïve PCa specimens, but also associated with early relapse after treatment. Recently, there has been considerable interest in the use of cell cycle-related genes as biomarkers of disease progression to aid treatment decisions. The cell cycle progression (CCP) test (Prolaris^®^, Myriad Genetics) is a prognostic assay based on a 46-gene expression signature that includes cell cycle-related genes, which, in combination with standard clinicopathological parameters, accurately stratifies patients with primary PCa to the risk of PCa-specific disease progression and disease-specific mortality [37]. Based on our preliminary findings, it is also possible that the CCP test may be useful to determine the risk of disease relapse after cytotoxic chemotherapy for advanced PCa.

Our study exemplifies the feasibility of *in vivo* RNA-Seq-based tumour molecular profiling from pre- and post-treatment biopsies from chemotherapy-treated patients [8] for advanced PCa to highlight the mechanisms of drug action and identify putative biomarkers of chemo-sensitivity or –resistance to (such as *ORM1*) and/or prognosis (such as *ADAM7* and *FAM72B*, and the cell cycle-related genes). Our preliminary findings suggest that a 7 gene signature biomarker panel, which includes cell-cycle related genes, may have prognostic value in treatment-naïve clinical PCa and warrants further investigation. Further similar larger-scale studies with high-quality outcomes data will be required to allow development of a complete oncogenomic personalised approach to patient care for advanced/metastatic PCa, with prognostication and treatment scheduling based on oncogenomic profiles to maximise chemotherapy efficacy [38].

## Conclusions

Here we report on the first “real-time” *in vivo* RNA-Seq-based transcriptome analysis of clinical PCa from pre- and post-treatment TRUSS-guided biopsies of patients treated with docetaxel chemotherapy plus ADT. We have identified a chemotherapy-driven PCa transcriptome profile which includes the down-regulation of important positive regulators of cell cycle progression. A 7-gene signature biomarker panel has been identified in high-risk prostate cancer patients to be of prognostic value. Future prospective study is warranted to evaluate the clinical value of this panel.

## Electronic supplementary material

Additional file 1: Table S1: Sequencing statistics and sample quality control. (XLSX 9 KB)

Additional file 2: Table S2: Fusion transcripts. Fusion transcripts expressed pre- and post-docetaxel plus ADT treatment arm identified by TopHat-Fusion. Identities and chromosomal loci of translocated genes are given. (XLSX 11 KB)

Additional file 3: Table S3: Differentially expressed genes. Differentially-expressed genes associated with docetaxel plus ADT (FDR < 0.05). (XLSX 46 KB)

Additional file 4: Figure S1: Survival analysis of patients with primary PCa (A) Kaplan Meier plot generated using cBioPortal [28 29] showing the survival curves of patients in the MSKCC Prostate Oncogenome Project dataset with and without alterations in expression of the top 6 differentially-expressed genes (Log_2_ fold change ≥ 2; FDR < 0.05) consistent in expression in at least 3 out of 4 patients (*p* < 0.05). (PDF 63 KB)

Additional file 5: Figure S2: Docetaxel-induced mitotic arrest occurs in the absence of androgens. **(A)** Ingenuity Pathway Analysis (IPA) showing the “Cell Cycle” network containing clusters of docetaxel and ADT-regulated genes. **(B)** Metacore canonical pathway map histograms after enrichment analysis of docetaxel and ADT-regulated genes **(C)** LNCaP cells were grown in full medium and subsequently transferred into steroid-depleted medium in the presence of docetaxel at 10 nM, 100 nM or 1 μM concentrations. After 48 hours of treatment, cells were harvested and stained with propidium iodide and subjected to cell cycle analysis by flow cytometry. Fold change in G2/M arrest LNCaP cell populations following docetaxel treatment at incremental doses. Data represent mean fold change +/− SEM from 3 independent biological experiments. (*Differences in the fold-change between conditions identified using the pooled-sample T-test with *p* < 0.05 taken to indicate statistical significance). **(D)** Matrix heatmap generated using cBioPortal [28, 29] showing alterations in expression of all 11 genes from within the KEGG term “Cell Cycle” in the MSKCC Prostate Oncogenome Project dataset [30]. (PDF 4 MB)

Additional file 6: Table S4: Enriched KEGG pathways. Pathways enriched at least 2-fold in genes either up or down regulated (FDR < 0.05). (XLS 20 KB)

Additional file 7: Table S5: Ingenuity Pathway Analysis (IPA). IPA analysis showing networks containing clusters of docetaxel and ADT-regulated genes (FDR < 0.05). (XLS 30 KB)

Additional file 8: Table S6: Enrichment for differentially expressed genes following docetaxel chemotherapy plus ADT within the KEGG pathway “Cell Cycle”. List of down-regulated genes enriched within the KEGG (Kyoto Encyclopedia of Genes and Genomes) [26] term “Cell Cycle” with at least 2-fold expression and FDR < 0.05. (FC = fold change). (XLSX 41 KB)

## Abbreviations

PCa: Prostate cancer
ADT: Androgen deprivation therapy
RNA-Seq: RNA-sequencing
TRUSS: Transrectal ultrasound
CRPCa: Castration resistant prostate cancer
KPS: Karnofsky performance status
EAU: European Association of Urology
FDR: False discovery rate
MSKCC: Memorial sloan kettering cancer centre
KEGG: Kyoto Encyclopedia of Genes and Genomes.
