# Supplementary figures and images for: Identification of a candidate prognostic gene signature by transcriptome analysis of matched pre- and post-treatment prostatic biopsies from patients with advanced prostate cancer
Source: BMC Cancer. 2014 Dec 18;14:977. doi: 10.1186/1471-2407-14-977 (PMC4301544; doi:10.1186/1471-2407-14-977)

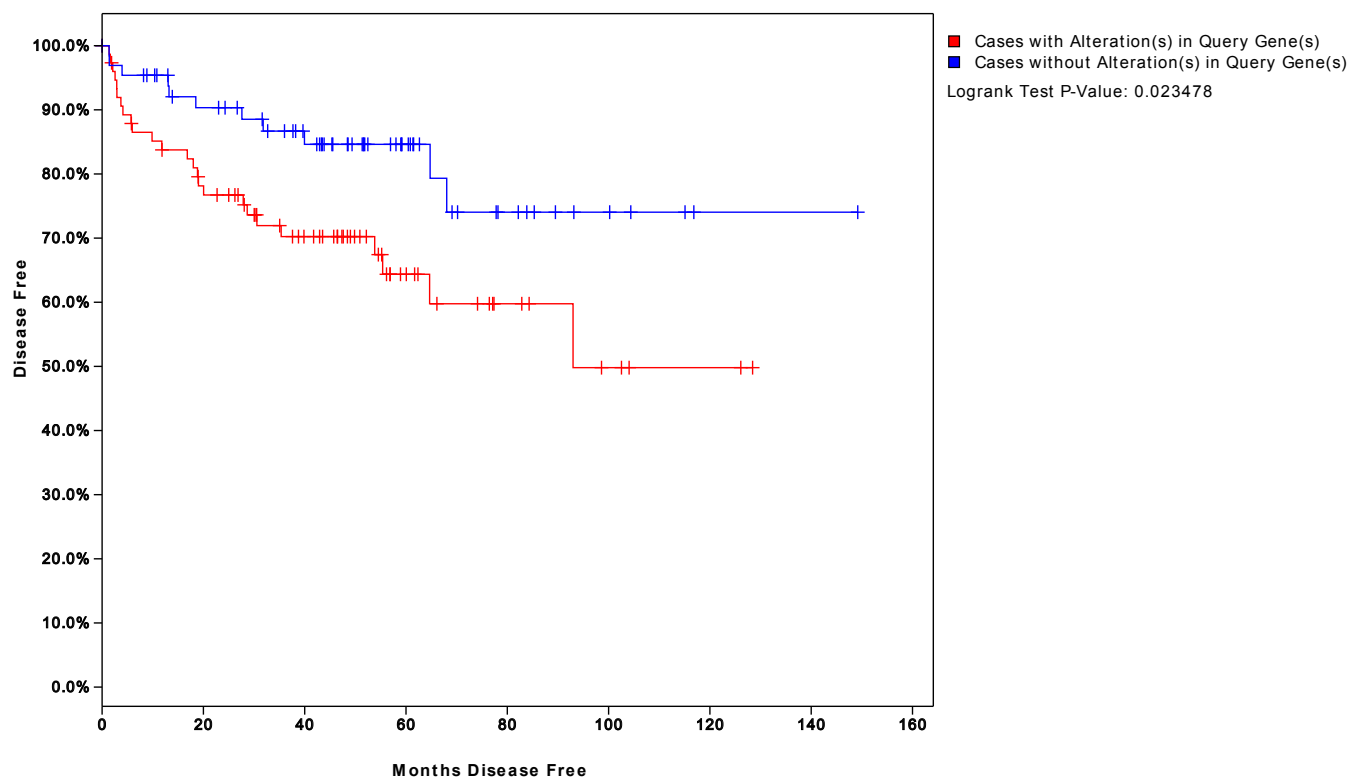

**Figure S1**

Supplement: Supplementary file 4 — Additional file 4: Figure S1: Survival analysis of patients with primary PCa (A) Kaplan Meier plot generated using cBioPortal [28 29] showing the survival curves of patients in the MSKCC Prostate Oncogenome Project dataset with and without alterations in expression of the top 6 differentially-expressed genes (Log2 fold change ≥ 2; FDR < 0.05) consistent in expression in at least 3 out of 4 patients (p < 0.05). (PDF 63 KB) [file 12885_2014_5118_MOESM4_ESM.pdf]

(A)

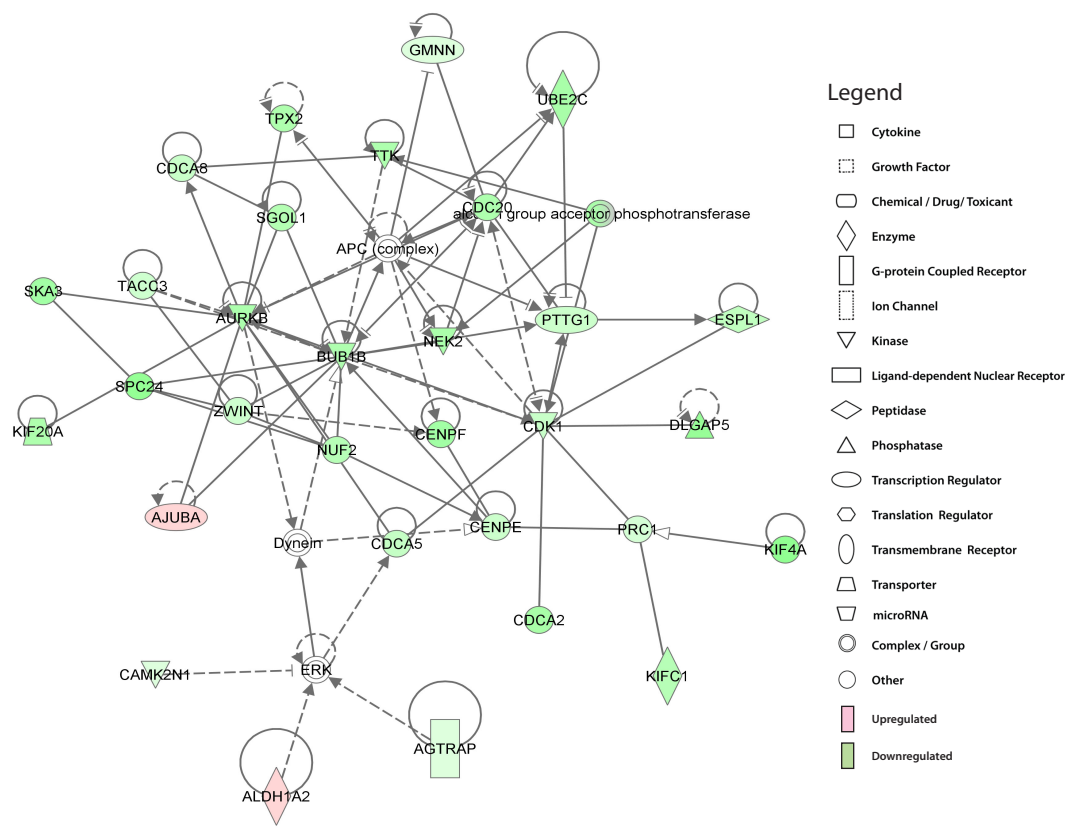

(B)

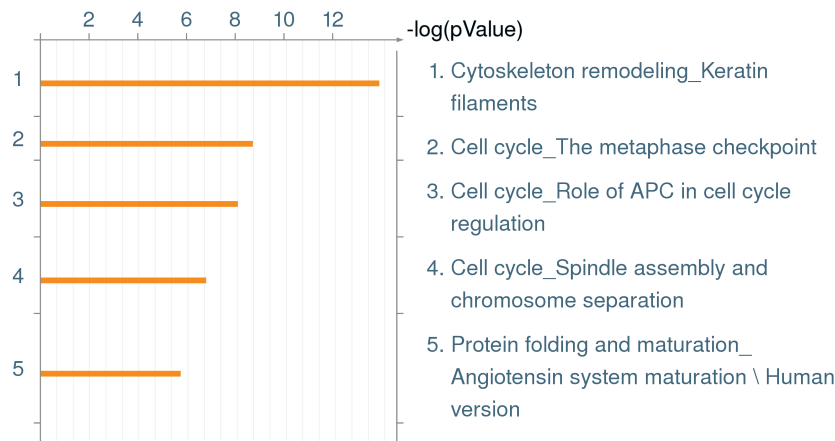

(C)

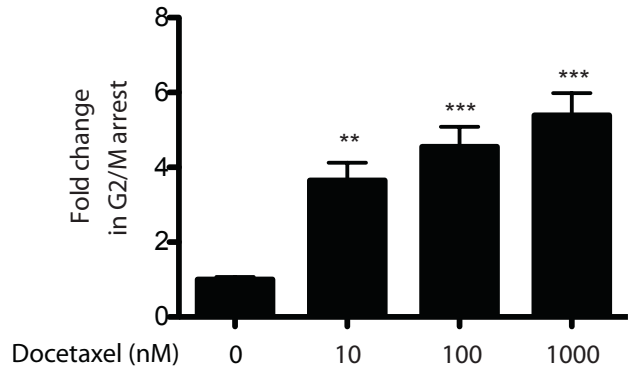

(D)

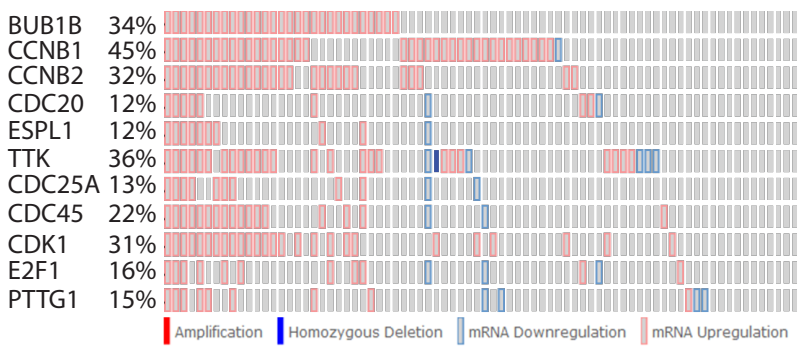

Figure S2

Supplement: Supplementary file 5 — Additional file 5: Figure S2: Docetaxel-induced mitotic arrest occurs in the absence of androgens. (A) Ingenuity Pathway Analysis (IPA) showing the “Cell Cycle” network containing clusters of docetaxel and ADT-regulated genes. (B) Metacore canonical pathway map histograms after enrichment analysis of docetaxel and ADT-regulated genes (C) LNCaP cells were grown in full medium and subsequently transferred into steroid-depleted medium in the presence of docetaxel at 10 nM, 100 nM or 1 μM concentrations. After 48 hours of treatment, cells were harvested and stained with propidium iodide and subjected to cell cycle analysis by flow cytometry. Fold change in G2/M arrest LNCaP cell populations following docetaxel treatment at incremental doses. Data represent mean fold change +/− SEM from 3 independent biological experiments. (*Differences in the fold-change between conditions identified using the pooled-sample T-test with p < 0.05 taken to indicate statistical significance). (D) Matrix heatmap generated using cBioPortal [28, 29] showing alterations in expression of all 11 genes from within the KEGG term “Cell Cycle” in the MSKCC Prostate Oncogenome Project dataset [30]. (PDF 4 MB) [file 12885_2014_5118_MOESM5_ESM.pdf]
